# Supplementary material for: Multi-Ancestry Transcriptome-Wide Association Studies of Cognitive Function, White Matter Hyperintensity, and Alzheimer’s Disease
Source: Int J Mol Sci. 2025 Mar 9;26(6):2443. doi: 10.3390/ijms26062443 (PMC11942532; doi:10.3390/ijms26062443)
Supplement: Supplementary file 1 [file ijms-26-02443-s001.zip › Chaar_Aim3_Supplementary_Figures_11.21.24.pdf]

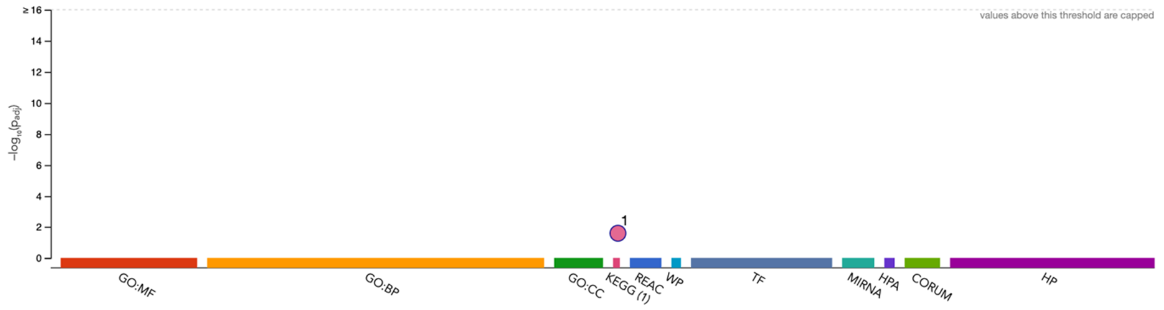

ID	Source	Term ID	Term Name	p_{adj} (query_1)
1	KEGG	KEGG:05010	Alzheimer disease	2.587×10^{-2}

Figure S1. Functional enrichment analysis on the gene set identified by METRO for general cognitive function and AD (N=22 genes; $P<2.90x10^{-6}$). The top panel consists of a Manhattan plot that illustrates the enrichment analysis results. The x-axis represents functional terms that are grouped and color-coded by data sources, including Gene Ontology (GO): molecular function (MF; red), GO: biological process (BP; orange), GO: cellular component (CC; dark green), Kyoto Encyclopedia of Genes and Genomes (KEGG; pink), Reactome (REAC; dark blue), WikiPathways (WP; turquoise), Transfac (TF; light blue), MiRTarBase (MIRNA; emerald green), Human Protein Atlas (HPA; dark purple), CORUM protein complexes (light green), and Human Phenotype Ontology (HP; violet), in order from left to right. The y-axis shows the adjusted enriched $-\log_{10}$ p-values <0.05. Multiple testing correction was performed using g:SCS method (Set Counts and Sizes) that takes into account overlapping terms. The top panel highlights driver GO terms identified using the greedy filtering algorithm in g:Profiler. The light circles represent terms that were not significant after filtering. The circle sizes are in accordance with the corresponding term size (i.e., larger terms have larger circles). The number in parentheses following the source name in the x-axis shows how many significantly enriched terms were from this source.

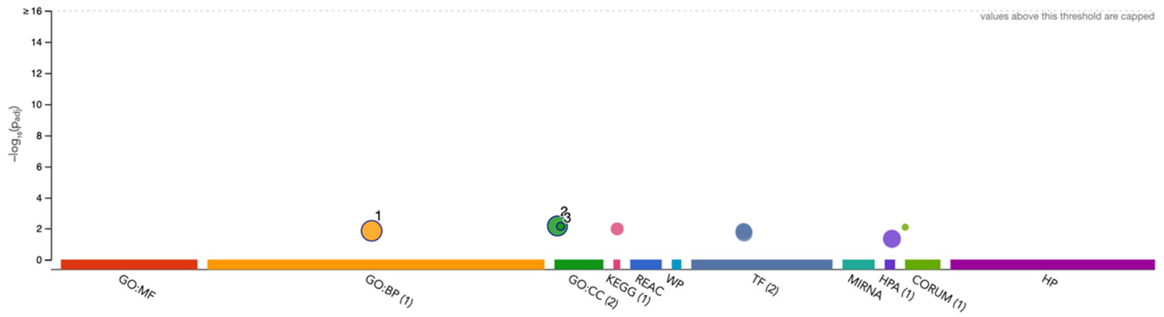

ID	Source	Term ID	Term Name	p_{adj} (query_1)
1	GO:BP	GO:0048518	positive regulation of biological process	1.429×10^{-2}
2	GO:CC	GO:0005654	nucleoplasm	6.986×10^{-3}
3	GO:CC	GO:0008537	proteasome activator complex	7.398×10^{-3}

Figure S2. Functional enrichment analysis on the fine-mapped gene set not previously identified by Davies et al. (2018)(1) **for general cognitive function using METRO TWAS (N=82 genes).** The top panel consists of a Manhattan plot that illustrates the enrichment analysis results. The x-axis represents functional terms that are grouped and color-coded by data sources, including Gene Ontology (GO): molecular function (MF; red), GO: biological process (BP; orange), GO: cellular component (CC; dark green), Kyoto Encyclopedia of Genes and Genomes (KEGG; pink), Reactome (REAC; dark blue), WikiPathways (WP; turquoise), Transfac (TF; light blue), MiRTarBase (MIRNA; emerald green), Human Protein Atlas (HPA; dark purple), CORUM protein complexes (light green), and Human Phenotype Ontology (HP; violet), in order from left to right. The y-axis shows the adjusted enriched $-\log_{10}$ p-values <0.05. Multiple testing correction was performed using g:SCS method (Set Counts and Sizes) that takes into account overlapping terms. The top panel highlights driver GO terms identified using the greedy filtering algorithm in g:Profiler. The light circles represent terms that were not significant after filtering. The circle sizes are in accordance with the corresponding term size (i.e., larger terms have larger circles). The number in parentheses following the source name in the x-axis shows how many significantly enriched terms were from this source.

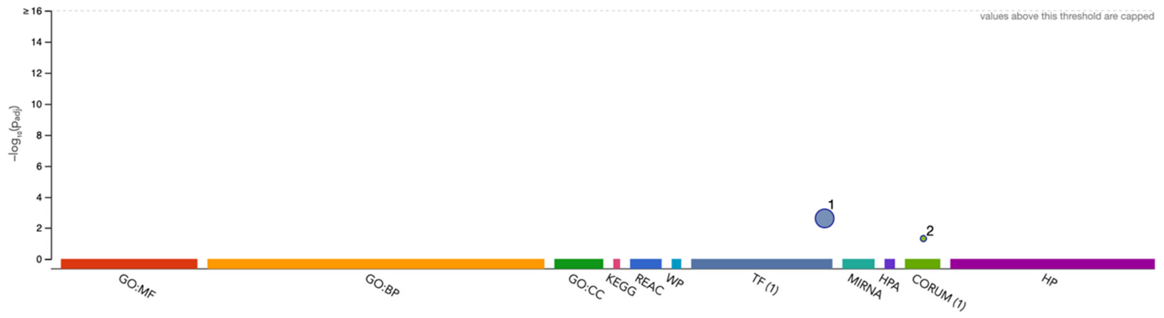

ID	Source	Term ID	Term Name	p_{adj} (query_1)
1	TF	TF:M12713	Factor: ZNF690; motif: GTCTACRCNG	2.520×10^{-3}
2	CORUM	CORUM:3991	ClpX complex, hexameric	4.968×10^{-2}

Figure S3. Functional enrichment analysis on the fine-mapped gene set not previously identified by Sargurupremraj et al. (2020)(2) for white matter hyperintensity using METRO TWAS (N=12 genes). The top panel consists of a Manhattan plot that illustrates the enrichment analysis results. The x-axis represents functional terms that are grouped and color-coded by data sources, including Gene Ontology (GO): molecular function (MF; red), GO: biological process (BP; orange), GO: cellular component (CC; dark green), Kyoto Encyclopedia of Genes and Genomes (KEGG; pink), Reactome (REAC; dark blue), WikiPathways (WP; turquoise), Transfac (TF; light blue), MiRTarBase (MIRNA; emerald green), Human Protein Atlas (HPA; dark purple), CORUM protein complexes (light green), and Human Phenotype Ontology (HP; violet), in order from left to right. The y-axis shows the adjusted enriched $-\log_{10}$ p-values <0.05. Multiple testing correction was performed using g:SCS method (Set Counts and Sizes) that takes into account overlapping terms. The top panel highlights driver GO terms identified using the greedy filtering algorithm in g:Profiler. The light circles represent terms that were not significant after filtering. The circle sizes are in accordance with the corresponding term size (i.e., larger terms have larger circles). The number in parentheses following the source name in the x-axis shows how many significantly enriched terms were from this source.

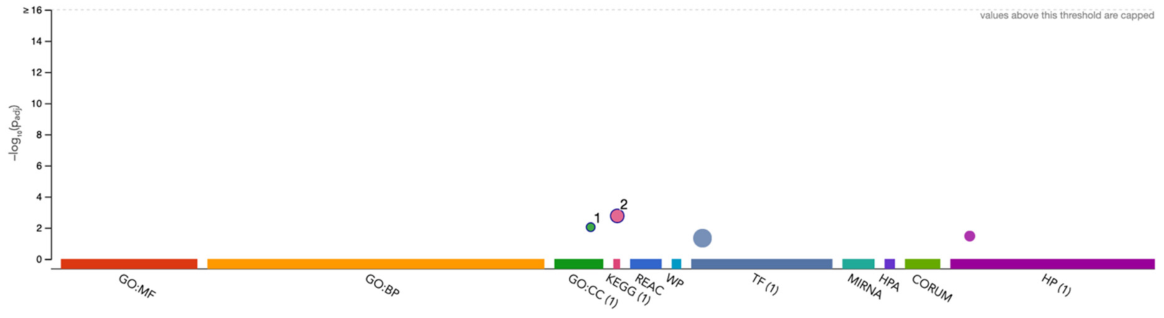

ID	Source	Term ID	Term Name	p_{adj} (query_1)
1	GO:CC	GO:0097418	neurofibrillary tangle	9.127×10^{-3}
2	KEGG	KEGG:04640	Hematopoietic cell lineage	1.729×10^{-3}

Figure S4. Functional enrichment analysis on the fine-mapped gene set not previously identified by Bellenguez et al. (2022)(3) for Alzheimer's disease using METRO TWAS (N=45 genes). The top panel consists of a Manhattan plot that illustrates the enrichment analysis results. The x-axis represents functional terms that are grouped and color-coded by data sources, including Gene Ontology (GO): molecular function (MF; red), GO: biological process (BP; orange), GO: cellular component (CC; dark green), Kyoto Encyclopedia of Genes and Genomes (KEGG; pink), Reactome (REAC; dark blue), WikiPathways (WP; turquoise), Transfac (TF; light blue), MiRTarBase (MIRNA; emerald green), Human Protein Atlas (HPA; dark purple), CORUM protein complexes (light green), and Human Phenotype Ontology (HP; violet), in order from left to right. The y-axis shows the adjusted enriched -$\log_{10}$ p-values <0.05. Multiple testing correction was performed using g:SCS method (Set Counts and Sizes) that takes into account overlapping terms. The top panel highlights driver GO terms identified using the greedy filtering algorithm in g:Profiler. The light circles represent terms that were not significant after filtering. The circle sizes are in accordance with the corresponding term size (i.e., larger terms have larger circles). The number in parentheses following the source name in the x-axis shows how many significantly enriched terms were from this source.

References

1. Davies G, Lam M, Harris SE, Trampush JW, Luciano M, Hill WD, et al. Study of 300,486 individuals identifies 148 independent genetic loci influencing general cognitive function. Nat Commun. 2018 May 29;9(1):2098.

2. Sargurupremraj M, Suzuki H, Jian X, Sarnowski C, Evans TE, Bis JC, et al. Cerebral small vessel disease genomics and its implications across the lifespan. Nature Communications. 2020 Dec 8;11(1):6285.

3. Bellenguez C, Küçükali F, Jansen IE, Kleineidam L, Moreno-Grau S, Amin N, et al. New insights into the genetic etiology of Alzheimer’s disease and related dementias. Nat Genet. 2022 Apr 4;1–25.
