# Supplementary material for: Multi-Ancestry Transcriptome-Wide Association Studies of Cognitive Function, White Matter Hyperintensity, and Alzheimer’s Disease
Source: Int J Mol Sci. 2025 Mar 9;26(6):2443. doi: 10.3390/ijms26062443 (PMC11942532; doi:10.3390/ijms26062443)
Supplement: Supplementary file 1 [file ijms-26-02443-s001.zip › Chaar_Aim3_Supplementary_Tables_02.19.25.pdf]

Table S1. Genes associated with general cognitive function using METRO followed by fine-mapping with FOCUS (N=266 genes; $P<2.9x10^{-6}$)

Gene	ENSG	alpha	w1	w2	P value	chr	Start	End
RNF123	ENSG00000164068	-0.23	0.58	0.42	3.20E-22	3	49726971	49758962
RABEP2	ENSG00000177548	-1.16	0.68	0.32	4.20E-22	16	28915742	28947847
GMPPB	ENSG00000173540	-0.28	1.00	0.00	8.35E-22	3	49754277	49761406
MST1	ENSG00000173531	-0.33	0.00	1.00	1.29E-21	3	49721380	49726934
APEH	ENSG00000164062	0.95	0.00	1.00	2.30E-21	3	49711447	49721404
IP6K1	ENSG00000176095	-0.76	0.62	0.38	4.32E-21	3	49761727	49823975
UBA7	ENSG00000182179	0.11	0.76	0.24	1.92E-20	3	49842642	49851386
TUFM	ENSG00000178952	-0.24	0.89	0.11	3.85E-20	16	28853732	28857669
FOXO6	ENSG00000204060	1.15	0.87	0.13	8.29E-20	1	41827594	41849262
NFKB2	ENSG00000077150	0.30	0.00	1.00	3.67E-19	10	104153867	104162281
PSD	ENSG00000059915	0.21	0.00	1.00	4.16E-19	10	104162374	104181296
STAU1	ENSG00000124214	0.17	0.52	0.48	9.53E-19	20	47729876	47804904
CSE1L	ENSG00000124207	-0.55	0.61	0.39	1.74E-18	20	47662783	47713497
ARFGEF2	ENSG00000124198	0.11	1.00	0.00	3.20E-18	20	47538248	47653230
USP4	ENSG00000114316	-0.76	0.27	0.73	4.14E-18	3	49314577	49378145
MEF2C	ENSG00000081189	-0.26	0.57	0.43	2.32E-17	5	88012934	88200074
PTPRD	ENSG00000153707	-0.70	1.00	0.00	1.03E-15	9	8314246	10613002
NEGR1	ENSG00000172260	-0.28	0.22	0.78	1.23E-15	1	71861626	72748222
SEMA3G	ENSG00000010319	-2.55	0.00	1.00	1.96E-15	3	52467051	52479119
ABT1	ENSG00000146109	0.61	0.39	0.61	2.24E-15	6	26597181	26600967
FOXP1	ENSG00000114861	1.10	0.93	0.07	3.34E-15	3	71003844	71633129
TET2	ENSG00000168769	-0.12	0.83	0.17	3.55E-15	4	106067032	106200973
ZNF322	ENSG00000181315	0.33	0.10	0.90	7.88E-15	6	26634611	26659980
HMGN4	ENSG00000182952	-0.09	0.00	1.00	1.08E-14	6	26538594	26547161
NISCH	ENSG00000010322	-0.25	1.00	0.00	5.24E-14	3	52489134	52527084
RBFOX1	ENSG00000078328	0.22	0.96	0.04	8.64E-14	16	5289803	7763342
ELAVL2	ENSG00000107105	-0.56	0.00	1.00	1.43E-13	9	23690102	23826335
IL27	ENSG00000197272	1.73	1.00	0.00	1.95E-13	16	28510683	28523372
TSNARE1	ENSG00000171045	-0.20	0.00	1.00	4.54E-13	8	143293441	143484543
KCNJ3	ENSG00000162989	0.34	0.00	1.00	5.53E-13	2	155554367	155714866
MTMR4	ENSG00000108389	-0.12	0.00	1.00	6.56E-13	17	56566890	56595266
AFF3	ENSG00000144218	-0.31	0.00	1.00	1.32E-12	2	100161881	100808890
LACE1	ENSG00000135537	-0.24	0.27	0.73	1.42E-12	6	108616195	108847999
ATXN1	ENSG00000124788	0.82	0.00	1.00	1.83E-12	6	16299343	16761722
PEF1	ENSG00000162517	0.11	0.79	0.21	2.25E-12	1	32095467	32110497
LONRF2	ENSG00000170500	0.45	0.00	1.00	3.21E-12	2	100888337	100938963
OR2J1	ENSG00000204702	-0.12	1.00	0.00	3.23E-12	6	29067267	29070478
HSF5	ENSG00000176160	-0.73	0.60	0.40	3.93E-12	17	56497528	56565769
ST3GAL3	ENSG00000126091	-0.09	0.90	0.10	5.88E-12	1	44171495	44396837
NKIRAS1	ENSG00000197885	-0.08	1.00	0.00	6.15E-12	3	23931442	23988082
COL16A1	ENSG00000084636	-1.07	0.00	1.00	6.99E-12	1	32117864	32169920
DCC	ENSG00000187323	-2.90	0.00	1.00	7.58E-12	18	49866567	51062273
FOXO3	ENSG00000118689	-1.70	0.00	1.00	1.09E-11	6	108881038	109005977
PRSS16	ENSG00000112812	0.30	0.53	0.47	1.11E-11	6	27215480	27224403
4-SEP	ENSG00000108387	-0.60	0.00	1.00	1.49E-11	17	56597611	56621729
QRICH1	ENSG00000198218	0.09	0.00	1.00	1.73E-11	3	49067140	49131796
RNF43	ENSG00000108375	-0.21	0.00	1.00	1.82E-11	17	56431037	56494956
ZNF193	ENSG00000137185	0.25	0.41	0.59	1.84E-11	6	28192664	28201265
ARF5	ENSG00000004059	0.15	1.00	0.00	2.40E-11	7	127228440	127231754
ZNF184	ENSG00000096654	0.21	1.00	0.00	2.54E-11	6	27418522	27440897
DPP4	ENSG00000197635	-1.30	0.02	0.98	2.70E-11	2	162848755	162930904
OR2J3	ENSG00000204701	-0.29	0.00	1.00	3.13E-11	6	29075835	29082547
SP4	ENSG00000105866	0.09	1.00	0.00	3.98E-11	7	21467661	21554440
FSCN3	ENSG00000106328	0.51	0.90	0.10	4.04E-11	7	127231463	127242198
GCC1	ENSG00000179562	0.13	0.88	0.12	4.72E-11	7	127220682	127233665
OR2H1	ENSG00000204688	-0.23	0.00	1.00	4.98E-11	6	29424932	29432105
SGCZ	ENSG00000185053	0.33	1.00	0.00	5.00E-11	8	13942354	15095940
FBXO41	ENSG00000163013	-0.21	0.99	0.01	5.10E-11	2	73481810	73511606

NR1D2	ENSG00000174738	-0.17	0.00	1.00	5.30E-11	3	23986777	24022108
DHODH	ENSG00000102967	-0.15	0.90	0.10	5.44E-11	16	72042487	72061563
HP	ENSG00000257017	-1.24	0.19	0.81	7.42E-11	16	72088404	72094954
ATF4	ENSG00000128272	-0.48	0.09	0.91	7.66E-11	22	39915700	39918688
TRIM27	ENSG00000204713	-0.92	0.00	1.00	9.36E-11	6	28870779	28891765
HIST1H2AG	ENSG00000196787	-0.17	0.00	1.00	1.00E-10	6	27100822	27101314
CCT7	ENSG00000135624	0.15	0.00	1.00	1.01E-10	2	73460548	73480149
THRB	ENSG00000151090	0.48	0.00	1.00	1.37E-10	3	24158644	24537247
ZKSCAN4	ENSG00000187626	0.42	0.23	0.77	1.44E-10	6	28209475	28220047
SND1	ENSG00000197157	-1.77	0.01	0.99	1.51E-10	7	127292248	127732661
PURA	ENSG00000185129	0.56	0.94	0.06	1.65E-10	5	139487362	139505204
SLC6A9	ENSG00000196517	0.25	0.37	0.63	1.71E-10	1	44457172	44497139
MGAT3	ENSG00000128268	0.38	0.00	1.00	1.91E-10	22	39853017	39888199
POU6F2	ENSG00000106536	0.55	0.00	1.00	1.92E-10	7	39017509	39532694
PPM1M	ENSG00000164088	0.12	0.58	0.42	2.21E-10	3	52279775	52284615
NPAS3	ENSG00000151322	0.72	1.00	0.00	2.44E-10	14	33403602	34290069
IST1	ENSG00000182149	0.17	0.00	1.00	2.75E-10	16	71879899	71965102
PRKAG1	ENSG00000181929	0.11	1.00	0.00	2.76E-10	12	49396057	49412590
HIST1H2BL	ENSG00000185130	-0.05	0.03	0.97	2.77E-10	6	27775257	27775707
PRADC1	ENSG00000135617	0.32	0.63	0.37	2.79E-10	2	73455138	73460367
CYSTM1	ENSG00000120306	0.35	0.00	1.00	2.82E-10	5	139554741	139661637
IPO9	ENSG00000198700	0.09	0.00	1.00	2.92E-10	1	201798277	201853419
EGR4	ENSG00000135625	0.95	0.87	0.13	3.14E-10	2	73518057	73520829
PDE4C	ENSG00000105650	-0.40	0.00	1.00	3.23E-10	19	18319462	18359010
HBEGF	ENSG00000113070	-0.50	0.00	1.00	3.38E-10	5	139712428	139726188
KIAA1683	ENSG00000130518	-0.19	0.00	1.00	3.52E-10	19	18367908	18385310
HPR	ENSG00000261701	-0.62	0.02	0.98	3.79E-10	16	72097047	72111145
NKAIN2	ENSG00000188580	24.44	0.00	1.00	5.73E-10	6	124125010	125146786
RNF39	ENSG00000204618	-0.53	0.66	0.34	5.95E-10	6	30038043	30043626
SRPK2	ENSG00000135250	-0.05	1.00	0.00	6.18E-10	7	104751151	105039755
SFXN5	ENSG00000144040	0.07	1.00	0.00	6.30E-10	2	73169165	73302747
MLL5	ENSG00000005483	0.25	1.00	0.00	6.40E-10	7	104581390	104755466
RBL2	ENSG00000103479	-0.11	0.00	1.00	6.89E-10	16	53467889	53525560
PFDN1	ENSG00000113068	0.16	0.76	0.24	7.66E-10	5	139624620	139682698
HIST1H2BJ	ENSG00000124635	0.04	1.00	0.00	7.95E-10	6	27093676	27100574
SPPL2C	ENSG00000185294	-0.82	0.41	0.59	8.29E-10	17	43922247	43924433
CDH8	ENSG00000150394	0.37	0.67	0.33	8.67E-10	16	61681146	62070939
FAM109B	ENSG00000177096	-0.13	1.00	0.00	1.03E-09	22	42470252	42475442
PTPRO	ENSG00000151490	-0.19	0.00	1.00	1.08E-09	12	15475191	15755109
SLC39A8	ENSG00000138821	0.18	0.00	1.00	1.35E-09	4	103172237	103352415
TNFRSF13C	ENSG00000159958	-0.01	0.00	1.00	1.37E-09	22	42318036	42322810
CPXM2	ENSG00000121898	-0.87	0.30	0.70	1.49E-09	10	125465723	125699783
PLCL1	ENSG00000115896	0.45	0.00	1.00	1.60E-09	2	198669317	199437305
NCOA2	ENSG00000140396	0.32	0.09	0.91	2.03E-09	8	71022017	71316043
PKD2L1	ENSG00000107593	0.26	0.70	0.30	2.28E-09	10	102047906	102090021
CDKAL1	ENSG00000145996	0.63	0.00	1.00	2.31E-09	6	20534688	21232635
SORT1	ENSG00000134243	-0.10	0.72	0.28	2.36E-09	1	109852190	109940540
TANK	ENSG00000136560	-0.43	0.69	0.31	2.37E-09	2	161993419	162092741
SUOX	ENSG00000139531	-0.08	0.57	0.43	2.38E-09	12	56390964	56400425
MLL2	ENSG00000167548	0.23	0.44	0.56	3.06E-09	12	49412758	49454577
LSM4	ENSG00000130520	0.43	0.55	0.45	4.06E-09	19	18417046	18433922
TIMM17A	ENSG00000134375	0.38	0.14	0.86	4.99E-09	1	201924631	201939792
RHEBL1	ENSG00000167550	-1.80	0.14	0.86	5.03E-09	12	49458459	49463808
CALN1	ENSG00000183166	-0.67	0.43	0.57	5.38E-09	7	71244476	71912136
PDE4D	ENSG00000113448	-0.19	1.00	0.00	6.24E-09	5	58264865	59817947
DDN	ENSG00000181418	-0.28	1.00	0.00	6.81E-09	12	49388932	49393158
CWF19L1	ENSG00000095485	0.07	0.00	1.00	7.48E-09	10	101992055	102027437
NKX2-1	ENSG00000136352	-0.42	0.56	0.44	8.23E-09	14	36985597	36990354
GDF15	ENSG00000130513	0.10	0.00	1.00	9.47E-09	19	18485541	18499986
NFIX	ENSG00000008441	0.24	0.03	0.97	1.02E-08	19	13106289	13209610
SNX29	ENSG00000048471	0.16	0.00	1.00	1.03E-08	16	12070591	12668144
AUTS2	ENSG00000158321	-0.25	0.00	1.00	1.16E-08	7	69063282	70258492

CHUK	ENSG00000213341	0.34	0.00	1.00	1.21E-08	10	101948057	101989353
RALYL	ENSG00000184672	-0.34	0.00	1.00	1.66E-08	8	85095022	85834079
PSMA5	ENSG00000143106	0.00	0.50	0.50	1.87E-08	1	109941664	109969070
PRKAR2B	ENSG00000005249	0.41	0.45	0.55	1.93E-08	7	106685150	106802256
EPS8	ENSG00000151491	-1.55	0.33	0.67	2.10E-08	12	15773068	16035263
LYL1	ENSG00000104903	0.25	0.00	1.00	2.25E-08	19	13209847	13213975
PSMC3	ENSG00000165916	-2.36	0.04	0.96	2.29E-08	11	47440320	47448024
LRRC14	ENSG00000160959	0.11	0.00	1.00	2.34E-08	8	145743376	145750556
WNT10B	ENSG00000169884	0.42	0.00	1.00	2.49E-08	12	49359123	49365518
RUNX1T1	ENSG00000079102	0.42	0.94	0.06	2.51E-08	8	92967195	93115514
PET112	ENSG00000059691	-0.05	0.90	0.10	3.00E-08	4	152591656	152682159
NKX2-8	ENSG00000136327	-0.20	0.00	1.00	3.10E-08	14	37049209	37051819
GLYCTK	ENSG00000168237	-0.13	0.67	0.33	3.20E-08	3	52321105	52329273
CAMK2N1	ENSG00000162545	0.18	0.59	0.41	3.50E-08	1	20808884	20812703
CKB	ENSG00000166165	0.10	0.12	0.88	3.68E-08	14	103986004	103989170
MYLK	ENSG00000065534	0.47	0.47	0.53	3.71E-08	3	123328896	123603179
CALR	ENSG00000179218	0.76	0.00	1.00	3.80E-08	19	13049392	13055303
JMJD1C	ENSG00000171988	0.30	0.31	0.69	4.49E-08	10	64926981	65281610
LRRC25	ENSG00000175489	0.13	1.00	0.00	4.61E-08	19	18501947	18508432
PPP1R16A	ENSG00000160972	0.31	0.45	0.55	4.66E-08	8	145703352	145727504
KCNJ6	ENSG00000157542	0.28	0.93	0.07	5.22E-08	21	38979675	39493439
EIF2B5	ENSG00000145191	0.96	0.45	0.55	5.93E-08	3	183852826	183863915
GADD45GIP1	ENSG00000179271	-0.41	1.00	0.00	5.97E-08	19	13063933	13068037
TONSL	ENSG00000160949	0.40	0.48	0.52	6.17E-08	8	145654158	145669823
ELK4	ENSG00000158711	-0.33	1.00	0.00	6.92E-08	1	205566684	205601139
NMNAT2	ENSG00000157064	1.60	0.76	0.24	7.81E-08	1	183217372	183387515
RAD23A	ENSG00000179262	0.16	1.00	0.00	7.92E-08	19	13056669	13064456
MACROD2	ENSG00000172264	-0.12	1.00	0.00	8.80E-08	20	13976015	16033842
DBN1	ENSG00000113758	0.07	1.00	0.00	9.91E-08	5	176883609	176901402
FAM193A	ENSG00000125386	0.09	0.10	0.90	1.04E-07	4	2538374	2734300
FARSA	ENSG00000179115	0.11	1.00	0.00	1.13E-07	19	13033293	13044851
NRBF2	ENSG00000148572	0.25	0.80	0.20	1.16E-07	10	64893007	64914791
ZSWIM6	ENSG00000130449	-1.09	0.00	1.00	1.18E-07	5	60628085	60841999
CCDC14	ENSG00000175455	-0.13	0.00	1.00	1.28E-07	3	123616152	123680255
CDH13	ENSG00000140945	-0.67	1.00	0.00	1.29E-07	16	82660570	83834245
MFSD4	ENSG00000174514	-0.07	1.00	0.00	1.31E-07	1	205538013	205572046
EPHA5	ENSG00000145242	0.29	0.00	1.00	1.53E-07	4	66185281	66536213
TSHZ3	ENSG00000121297	-0.79	0.55	0.45	1.54E-07	19	31640885	31840342
SLC39A4	ENSG00000147804	-1.75	0.00	1.00	1.65E-07	8	145635126	145642228
DAND5	ENSG00000179284	1.23	0.00	1.00	1.66E-07	19	13075973	13085574
PDCL3	ENSG00000115539	-0.11	1.00	0.00	1.79E-07	2	101179455	101193201
DCAF11	ENSG00000100897	-0.77	0.03	0.97	1.84E-07	14	24583404	24594451
GCDH	ENSG00000105607	0.18	1.00	0.00	1.96E-07	19	13001974	13025021
TMEM180	ENSG00000138111	-0.09	1.00	0.00	2.03E-07	10	104221152	104236802
AGAP1	ENSG00000157985	-0.62	0.33	0.67	2.35E-07	2	236402687	237040444
KLF1	ENSG00000105610	-1.72	0.18	0.82	2.49E-07	19	12995236	12998015
CDH4	ENSG00000179242	0.36	0.51	0.49	2.63E-07	20	59827317	60515673
CFB	ENSG00000243649	-1.58	0.93	0.07	2.64E-07	6	31913427	31919861
MAML2	ENSG00000184384	0.24	0.00	1.00	2.71E-07	11	95709762	96076359
IL34	ENSG00000157368	0.44	0.86	0.14	2.75E-07	16	70613798	70694585
CPEB1	ENSG00000214575	0.12	0.00	1.00	2.90E-07	15	83211951	83317612
MTSS1L	ENSG00000132613	0.48	1.00	0.00	2.95E-07	16	70695107	70719956
GRK6	ENSG00000198055	-0.23	0.67	0.33	3.02E-07	5	176830205	176869902
IL17D	ENSG00000172458	0.41	0.50	0.50	3.23E-07	13	21276266	21297237
FMNL3	ENSG00000161791	-0.26	0.44	0.56	3.28E-07	12	50030282	50101948
RORA	ENSG00000069667	0.25	0.01	0.99	3.44E-07	15	60780483	61521501
FBXL17	ENSG00000145743	0.37	0.77	0.23	3.45E-07	5	107194736	107717799
NCAM1	ENSG00000149294	-0.89	0.72	0.28	3.58E-07	11	112831969	113149158
CSRNP3	ENSG00000178662	0.34	0.53	0.47	3.60E-07	2	166326157	166545917
SSBP2	ENSG00000145687	0.42	0.63	0.37	3.82E-07	5	80708623	81047616
CPNE6	ENSG00000100884	0.26	1.00	0.00	4.05E-07	14	24540046	24547309
FITM1	ENSG00000139914	-0.42	0.00	1.00	4.07E-07	14	24599868	24602058

SEMA3E	ENSG00000170381	0.20	0.16	0.84	4.08E-07	7	82992554	83278455
NDUFAF2	ENSG00000164182	-0.17	1.00	0.00	4.27E-07	5	60241004	60450358
LDB2	ENSG00000169744	0.23	0.00	1.00	4.45E-07	4	16503164	16900301
MGST2	ENSG00000085871	0.07	0.76	0.24	4.92E-07	4	140586922	140661899
RNF4	ENSG00000063978	0.05	0.29	0.71	5.04E-07	4	2463947	2627047
RAI1	ENSG00000108557	-0.08	0.00	1.00	5.43E-07	17	17584772	17714767
IGSF9B	ENSG00000080854	0.26	1.00	0.00	5.50E-07	11	133766333	133826863
ANKS1B	ENSG00000185046	-0.26	0.70	0.30	5.51E-07	12	99120235	100378714
C6orf108	ENSG00000112667	-0.05	0.75	0.25	5.66E-07	6	43193367	43197219
C6orf25	ENSG00000204420	-0.18	0.20	0.80	5.80E-07	6	31686371	31694491
CDH9	ENSG00000113100	-0.28	1.00	0.00	6.16E-07	5	26880706	27121257
TNRC6A	ENSG00000090905	-0.14	1.00	0.00	6.19E-07	16	24621530	24838953
CRAT	ENSG00000095321	-0.03	0.00	1.00	6.32E-07	9	131856421	131873468
C18orf1	ENSG00000168675	0.69	1.00	0.00	6.32E-07	18	13217497	13652754
C17orf59	ENSG00000196544	-0.35	0.55	0.45	6.45E-07	17	8091663	8093498
SLC34A1	ENSG00000131183	-0.33	1.00	0.00	6.89E-07	5	176806236	176825849
CEP192	ENSG00000101639	0.03	0.10	0.90	6.97E-07	18	12991361	13125051
FBXL4	ENSG00000112234	-0.11	1.00	0.00	7.02E-07	6	99316411	99395882
NFAM1	ENSG00000235568	0.17	0.00	1.00	7.09E-07	22	42776413	42828409
C20orf173	ENSG00000125975	-0.19	0.70	0.30	7.20E-07	20	34111014	34117481
PRR7	ENSG00000131188	0.51	0.00	1.00	7.29E-07	5	176873446	176883287
SCN2A	ENSG00000136531	0.14	0.00	1.00	8.00E-07	2	166051503	166248820
SP2	ENSG00000167182	0.16	0.00	1.00	8.10E-07	17	45973516	46006323
CPD	ENSG00000108582	-0.08	0.55	0.45	8.37E-07	17	28705945	28797007
CRIP3	ENSG00000146215	0.31	1.00	0.00	8.49E-07	6	43267448	43276564
SHISA9	ENSG00000237515	-0.53	0.28	0.72	8.58E-07	16	12995455	13334273
SEMA6D	ENSG00000137872	-0.76	1.00	0.00	8.84E-07	15	47476298	48066425
XXbac-BPG32J3.19	ENSG00000250641	0.27	0.71	0.29	8.98E-07	6	31674681	31685695
MAML3	ENSG00000196782	0.15	0.88	0.12	9.55E-07	4	140637907	141075338
SMG7	ENSG00000116698	0.00	0.50	0.50	9.78E-07	1	183441351	183567381
SLC22A7	ENSG00000137204	-0.21	0.00	1.00	1.03E-06	6	43263432	43273276
CADM2	ENSG00000175161	-0.11	0.82	0.18	1.04E-06	3	85008140	86123579
PSME2	ENSG00000100911	-0.72	0.04	0.96	1.04E-06	14	24612571	24616779
GPD2	ENSG00000115159	0.20	1.00	0.00	1.13E-06	2	157291802	157470247
PTPRT	ENSG00000196090	-0.66	0.22	0.78	1.16E-06	20	40701392	41818610
AKAP6	ENSG00000151320	0.00	0.50	0.50	1.17E-06	14	32798504	33306890
MTMR2	ENSG00000087053	1.64	0.00	1.00	1.18E-06	11	95554930	95658479
C19orf81	ENSG00000235034	-0.61	0.62	0.38	1.22E-06	19	51152702	51162567
RMI1	ENSG00000178966	-0.28	0.63	0.37	1.28E-06	9	86595713	86618989
CALML5	ENSG00000178372	0.37	0.01	0.99	1.28E-06	10	5540660	5541533
ATP5H	ENSG00000167863	6.63	0.02	0.98	1.28E-06	17	73034958	73043080
TMBIM6	ENSG00000139644	0.05	1.00	0.00	1.32E-06	12	50101508	50158717
RGSL1	ENSG00000121446	-0.14	1.00	0.00	1.34E-06	1	182378327	182529734
AVL9	ENSG00000105778	-0.08	0.00	1.00	1.38E-06	7	32535038	32628338
TYW5	ENSG00000162971	0.06	0.42	0.58	1.38E-06	2	200793636	200820459
PSME1	ENSG00000092010	-1.04	0.00	1.00	1.49E-06	14	24605372	24608176
KCNK3	ENSG00000171303	1.06	0.36	0.64	1.51E-06	2	26915590	26956288
HSPA1A	ENSG00000204389	-0.48	0.06	0.94	1.65E-06	6	31783320	31785723
LMF1	ENSG00000103227	-0.04	0.93	0.07	1.71E-06	16	903634	1031318
MCRS1	ENSG00000187778	-0.04	1.00	0.00	1.76E-06	12	49950327	49961928
SPAG4	ENSG00000061656	-0.12	0.25	0.75	1.82E-06	20	34203751	34209016
SLC5A11	ENSG00000158865	0.18	1.00	0.00	1.94E-06	16	24857162	24922949
EXT1	ENSG00000182197	-0.09	0.00	1.00	1.94E-06	8	118806729	119124065
MAST4	ENSG00000069020	0.11	0.97	0.03	1.94E-06	5	65892208	66465421
ROMO1	ENSG00000125995	-0.34	0.37	0.63	1.97E-06	20	34287194	34288906
TMEM170B	ENSG00000205269	-0.06	0.81	0.19	1.98E-06	6	11537982	11583757
RPS17L	ENSG00000182774	0.23	0.83	0.17	2.00E-06	15	83205501	83209210
LRRC9	ENSG00000131951	-0.14	0.00	1.00	2.01E-06	14	60386431	60530277
ZNF638	ENSG00000075292	-0.11	0.00	1.00	2.04E-06	2	71503691	71662199
C9orf64	ENSG00000165118	-0.07	1.00	0.00	2.05E-06	9	86553226	86571901
SH3RF3	ENSG00000172985	0.08	0.83	0.17	2.09E-06	2	109745661	110262211
BCL11A	ENSG00000119866	-0.28	0.58	0.42	2.11E-06	2	60677655	60781602

MACROD1	ENSG00000133315	0.21	1.00	0.00	2.14E-06	11	63766030	63933585
PURG	ENSG00000172733	0.15	0.14	0.86	2.15E-06	8	30853318	30891231
RP11-468E2.4	ENSG00000259529	-2.84	0.00	1.00	2.15E-06	14	24616757	24635661
CUL9	ENSG00000112659	0.20	0.29	0.71	2.21E-06	6	43149922	43192325
LARGE	ENSG00000133424	-0.18	0.92	0.08	2.21E-06	22	33558212	34318829
VEGFA	ENSG00000112715	0.18	0.12	0.88	2.21E-06	6	43737921	43754224
PHF20	ENSG00000025293	-0.10	0.99	0.01	2.25E-06	20	34359896	34538292
SETBP1	ENSG00000152217	0.25	0.89	0.11	2.25E-06	18	42260138	42648475
C2orf47	ENSG00000162972	0.31	0.48	0.52	2.29E-06	2	200820040	200873263
KBTBD2	ENSG00000170852	-0.90	0.00	1.00	2.48E-06	7	32907784	32933743
CNGB3	ENSG00000170289	3.10	1.00	0.00	2.56E-06	8	87566205	87755903
PCK2	ENSG00000100889	0.35	0.00	1.00	2.56E-06	14	24563262	24579807
DCAF5	ENSG00000139990	0.16	1.00	0.00	2.64E-06	14	69517598	69619867
PRPF38A	ENSG00000134748	0.20	0.17	0.83	2.67E-06	1	52870274	52886508
SREBF1	ENSG00000072310	-0.35	0.41	0.59	2.67E-06	17	17713713	17740316
NCKAP5L	ENSG00000167566	0.40	0.75	0.25	2.67E-06	12	50184929	50222533
FAM76B	ENSG00000077458	0.10	1.00	0.00	2.72E-06	11	95502117	95523573
MED27	ENSG00000160563	0.20	1.00	0.00	2.74E-06	9	134728315	134955254
CPNE3	ENSG00000085719	0.21	0.63	0.37	2.74E-06	8	87526664	87573726
SYT3	ENSG00000213023	-0.58	0.27	0.73	2.82E-06	19	51124564	51143138
DCDC2	ENSG00000146038	0.12	1.00	0.00	2.84E-06	6	24171983	24358287
CHCHD3	ENSG00000106554	-0.11	0.31	0.69	2.89E-06	7	132469631	132766850

Abbreviations: ENSG, Ensembl gene ID; alpha, overall effect of the GreX; w1, contribution weight for African ancestry; w2, contribution weight for European ancestry; chr, chromosome.

Table S2. Genes associated with white matter hyperintensity using METRO followed by fine-mapping with FOCUS (N=23 genes; $P<2.9x10^{-6}$)

Gene	ENSG	alpha	w1	w2	P value	chr	Start	End
WBP2	ENSG00000132471	1.52	0.00	1.00	7.92E-54	17	73841780	73852588
SH3PXD2A	ENSG00000107957	0.00	0.50	0.50	5.78E-20	10	105353784	105615342
DCAKD	ENSG00000172992	3.93	0.00	1.00	3.13E-19	17	43100706	43138499
NMT1	ENSG00000136448	-0.44	0.00	1.00	1.41E-16	17	43035360	43186384
EFEMP1	ENSG00000115380	0.89	1.00	0.00	1.35E-15	2	56093102	56151274
ICA1L	ENSG00000163596	-0.27	0.36	0.64	5.44E-12	2	203637873	203736489
NBEAL1	ENSG00000144426	0.57	0.00	1.00	4.91E-11	2	203879331	204091101
WDR12	ENSG00000138442	-0.64	0.00	1.00	5.73E-11	2	203738984	203879521
KLHL24	ENSG00000114796	-0.99	0.00	1.00	1.01E-10	3	183353398	183402307
NEURL	ENSG00000107954	0.93	0.44	0.56	3.28E-10	10	105253462	105352303
CALCRL	ENSG00000064989	1.08	0.94	0.06	7.96E-10	2	188206691	188313187
HAAO	ENSG00000162882	-0.24	1.00	0.00	1.18E-08	2	42994229	43019733
ARMS2	ENSG00000254636	-0.62	0.00	1.00	1.64E-08	10	124214169	124216868
GJC1	ENSG00000182963	-0.06	0.00	1.00	3.57E-08	17	42875816	42908184
OXER1	ENSG00000162881	0.53	1.00	0.00	7.02E-08	2	42989639	42991275
HTRA1	ENSG00000166033	7.07	1.00	0.00	2.53E-07	10	124218067	124274423
LRRC37A3	ENSG00000176809	-0.44	0.00	1.00	2.54E-07	17	62850248	62915598
FBXO31	ENSG00000103264	1.11	0.00	1.00	3.05E-07	16	87360593	87425748
PDCD7	ENSG00000090470	-1.81	0.00	1.00	3.54E-07	15	65409717	65426146
CLPX	ENSG00000166855	0.40	1.00	0.00	3.54E-07	15	65440557	65477680
EFTUD2	ENSG00000108883	0.00	0.50	0.50	4.49E-07	17	42927316	42976813
UBAP1L	ENSG00000246922	-1.26	0.01	0.99	5.91E-07	15	65385098	65407538
MAP1LC3B	ENSG00000140941	0.37	0.13	0.87	1.94E-06	16	87417559	87438385

Abbreviations: ENSG, Ensembl gene ID; alpha, overall effect of the GreX; w1, contribution weight for African ancestry; w2, contribution weight for European ancestry; chr, chromosome.

Table S3. Genes associated with Alzheimer's disease (EA GWAS) using METRO followed by fine-mapping with FOCUS (N=69 genes; $P<2.9x10^{-6}$)

Gene	ENSG	alpha	w1	w2	P value	chr	Start	End
CLU	ENSG00000120885	0.48	0.29	0.71	4.77E-39	8	27454434	27472217
TOMM40	ENSG00000130204	-19.78	1.00	0.00	4.77E-39	19	45393826	45406946
APOE	ENSG00000130203	67.11	0.28	0.72	4.77E-39	19	45409048	45412650
APOC4	ENSG00000224916	-17.64	0.91	0.09	4.77E-39	19	45445495	45452822
PICALM	ENSG00000073921	-0.15	1.00	0.00	9.33E-36	11	85668218	85780924
CCDC83	ENSG00000150676	0.28	1.00	0.00	1.01E-26	11	85566144	85631064
CR2	ENSG00000117322	1.26	0.00	1.00	1.87E-25	1	207627575	207663240
MS4A6A	ENSG00000110077	-0.29	0.58	0.42	1.48E-19	11	59939488	59952139
MS4A2	ENSG00000149534	1.51	0.22	0.78	2.29E-17	11	59855734	59865940
RIN3	ENSG00000100599	3.19	1.00	0.00	7.51E-17	14	92980125	93155339
MEPCE	ENSG00000146834	-0.22	1.00	0.00	5.62E-16	7	100025945	100031749
PPP1R35	ENSG00000160813	0.31	1.00	0.00	2.40E-15	7	100032905	100034120
CYB561	ENSG00000008283	0.12	0.85	0.15	1.66E-14	17	61509665	61523715
KCNH6	ENSG00000173826	-0.27	0.62	0.38	2.33E-14	17	61600695	61626338
USP6	ENSG00000129204	-0.38	0.98	0.02	3.47E-14	17	5019327	5078329
TREM2	ENSG00000095970	1.10	0.00	1.00	7.75E-14	6	41126244	41130924
ACE	ENSG00000159640	0.65	0.24	0.76	8.53E-14	17	61554422	61575741
ZYX	ENSG00000159840	0.17	0.00	1.00	9.46E-14	7	143078388	143088204
FAM131B	ENSG00000159784	-0.28	1.00	0.00	2.78E-13	7	143050493	143059863
C6orf10	ENSG00000204296	-0.39	0.00	1.00	3.04E-13	6	32256303	32339689
BTNL2	ENSG00000204290	0.60	0.76	0.24	7.66E-13	6	32361116	32374958
ATG16L1	ENSG00000085978	-0.12	0.00	1.00	1.33E-12	2	234118697	234204320
TREML1	ENSG00000161911	-1.07	0.96	0.04	2.31E-12	6	41117075	41122085
ZNF594	ENSG00000180626	0.98	0.40	0.60	2.44E-12	17	5082830	5095163
EPHA1	ENSG00000146904	-0.58	0.52	0.48	3.79E-12	7	143087382	143105949
APP	ENSG00000142192	-0.13	0.00	1.00	1.09E-11	21	27252861	27543446
USP6NL	ENSG00000148429	0.08	0.40	0.60	1.33E-10	10	11502509	11653665
SLC39A13	ENSG00000165915	-0.12	0.00	1.00	2.95E-10	11	47428683	47438047
PPP4C	ENSG00000149923	0.03	0.92	0.08	3.65E-10	16	30087299	30096697
SLC52A1	ENSG00000132517	0.78	0.27	0.73	3.69E-10	17	4935895	4955304
AC008394.1	ENSG00000233828	0.17	0.00	1.00	5.07E-10	5	86512423	86534822
FAM210B	ENSG00000124098	-0.14	0.00	1.00	9.14E-10	20	54934030	54943719
GCNT7	ENSG00000124091	-3.29	0.98	0.02	9.16E-10	20	55066548	55100981
RAPSN	ENSG00000165917	0.14	0.99	0.01	1.37E-09	11	47459315	47470695
CD55	ENSG00000196352	-0.47	1.00	0.00	1.84E-09	1	207494864	207560149
HLA-DQA2	ENSG00000237541	-0.06	0.00	1.00	1.87E-09	6	32709168	32714975
TBX6	ENSG00000149922	-0.11	0.96	0.04	1.95E-09	16	30097114	30103245
DYDC2	ENSG00000133665	-0.44	0.33	0.67	2.42E-09	10	82104501	82127829
CSTF1	ENSG00000101138	-4.15	0.00	1.00	2.62E-09	20	54967427	54981418
CASS4	ENSG00000087589	-0.18	0.37	0.63	4.41E-09	20	54987168	55035443
DYDC1	ENSG00000170788	0.30	0.39	0.61	4.65E-09	10	82095861	82116511
LILRA6	ENSG00000244482	0.21	0.13	0.87	5.48E-09	19	54720737	54746649
MME	ENSG00000196549	-0.23	1.00	0.00	5.99E-09	3	154741913	154901493
NIT1	ENSG00000158793	-0.24	0.32	0.68	7.12E-09	1	161087876	161095235
KLF16	ENSG00000129911	0.18	1.00	0.00	1.03E-08	19	1852398	1863578
DEDD	ENSG00000158796	0.15	0.00	1.00	1.17E-08	1	161090764	161102478
MAF1	ENSG00000179632	-0.11	0.00	1.00	1.52E-08	8	145159364	145162514
TP53INP1	ENSG00000164938	-0.05	0.66	0.34	2.05E-08	8	95938200	95961606
KANSL1	ENSG00000120071	0.00	0.50	0.50	3.81E-08	17	44107282	44302755
LACTB	ENSG00000103642	0.06	0.41	0.59	4.42E-08	15	63414032	63434260
SHARPIN	ENSG00000179526	0.23	1.00	0.00	9.56E-08	8	145153536	145163027
MAPT	ENSG00000186868	-0.54	0.51	0.49	1.76E-07	17	43971893	44105700

IKZF1	ENSG00000185811	-0.13	1.00	0.00	3.33E-07	7	50343664	50472799
CPSF3	ENSG00000119203	0.19	1.00	0.00	3.85E-07	2	9563780	9613230
CCNE2	ENSG00000175305	-0.18	0.35	0.65	4.65E-07	8	95891998	95908906
FAM108A1	ENSG00000129968	-0.81	0.68	0.32	4.69E-07	19	1876809	1885495
GRN	ENSG00000030582	0.18	0.84	0.16	6.31E-07	17	42422614	42430474
ABI3	ENSG00000108798	-0.05	0.00	1.00	9.18E-07	17	47287773	47300587
SLTM	ENSG00000137776	0.17	1.00	0.00	9.82E-07	15	59171249	59225878
RNF111	ENSG00000157450	-0.39	0.06	0.94	1.04E-06	15	59157374	59389618
EPDR1	ENSG00000086289	0.04	1.00	0.00	1.16E-06	7	37723446	37991538
SUPT4H1	ENSG00000213246	0.06	0.76	0.24	1.27E-06	17	56422536	56430454
STYX	ENSG00000198252	0.10	0.00	1.00	1.38E-06	14	53196884	53241707
SIGLEC9	ENSG00000129450	-0.17	0.00	1.00	1.82E-06	19	51628163	51639908
DDX54	ENSG00000123064	0.22	0.36	0.64	2.02E-06	12	113594978	113623284
TRIB1	ENSG00000173334	0.42	0.00	1.00	2.41E-06	8	126442600	126450645
BLNK	ENSG00000095585	0.40	0.00	1.00	2.55E-06	10	97948927	98031344
SLC25A39	ENSG00000013306	-0.58	1.00	0.00	2.57E-06	17	42396993	42402238
ICA1L	ENSG00000163596	-0.06	0.41	0.59	2.68E-06	2	203637873	203736489

Abbreviations: EA, European ancestry; ENSG, Ensembl gene ID; alpha, overall effect of the GreX; w1, contribution weight for African ancestry; w2, contribution weight for European ancestry; chr, chromosome.

Table S4. Genes associated with Alzheimer's disease (AA GWAS) using METRO followed by fine-mapping with FOCUS (N=2 genes; $P<2.9x10^{-6}$)

Gene	ENSG	alpha	w1	w2	P value	chr	Start	End
PVRL2	ENSG00000130202	-10.72	0.55221657	0.44778343	9.67E-81	19	45349393	45392485
TOMM40	ENSG00000130204	10.02	0.04436227	0.95563773	1.15E-80	19	45394477	45406935

Abbreviations: AA, African ancestry; ENSG, Ensembl gene ID; alpha, overall effect of the GreX; w1, contribution weight for African ancestry; w2, contribution weight for European ancestry; chr, chromosome.
